# RETRACTION: Knee and Hip Arthroplasty Joint Surgical Site Wound Infection in End‐Stage Renal Disease Subjects Who Underwent Dialysis or a Kidney Transplant: A Meta‐Analysis

**DOI:** 10.1111/iwj.70216

**Published:** 2025-02-11

## Abstract

**RETRACTION**: LuoY. , GongJ. , and YangS. , “Knee and Hip Arthroplasty Joint Surgical Site Wound Infection in End‐Stage Renal Disease Subjects Who Underwent Dialysis or a Kidney Transplant: A Meta‐Analysis,” International Wound Journal 20, no. 7 (2023): 2811–2819, 10.1111/iwj.14160.37038328 PMC10410356

The above article, published online on 10 April 2023, in Wiley Online Library (http://onlinelibrary.wiley.com/), has been retracted by agreement between the journal Editor in Chief, Professor Keith Harding; and John Wiley & Sons Ltd. Following an investigation by the publisher, all parties have concluded that this article was accepted solely on the basis of a compromised peer review process. The editors have therefore decided to retract the article. The authors did not respond to our notice regarding the retraction.

**RETRACTION**: Y. Luo , J. Gong , and S. Yang , “Knee and Hip Arthroplasty Joint Surgical Site Wound Infection in End‐Stage Renal Disease Subjects Who Underwent Dialysis or a Kidney Transplant: A Meta‐Analysis,” International Wound Journal 20, no. 7 (2023): 2811–2819, 10.1111/iwj.14160.37038328 PMC10410356

The above article, published online on 10 April 2023, in Wiley Online Library (http://onlinelibrary.wiley.com/), has been retracted by agreement between the journal Editor in Chief, Professor Keith Harding; and John Wiley & Sons Ltd. Following an investigation by the publisher, all parties have concluded that this article was accepted solely on the basis of a compromised peer review process. The editors have therefore decided to retract the article. The authors did not respond to our notice regarding the retraction.

